# Dietary Patterns and Healthy Habits Along the Life Course

**DOI:** 10.5935/abc.20190127

**Published:** 2019-07

**Authors:** Lucia Campos Pellanda

**Affiliations:** 1 Universidade Federal de Ciências da Saude de Porto Alegre - Saúde Coletiva, Porto Alegre, RS - Brazil; 2 Instituto de Cardiologia / Fundação Universitária de Cardiologia (IC/FUC), Porto Alegre, RS - Brazil

**Keywords:** Healthy Diet, Diet Western, Risk Reduction Behavior, Aculturation, Exercise, Social Class, Multiple Chronic Conditions

Childhood represents a critical window in the life course for the establishment of dietary patterns and other healthy habits. In the last decades, these habits have been changing significantly, and childhood obesity has become an increasing public health issue, with many clinical consequences.^1-3^

To address these issues, it is of paramount importance to understand dietary patterns and their relation to body measurements, as Rocha et al propose in the paper “Association of Dietary Patterns with Excess Weight and Body Adiposity in Brazilian Children: The Pase-Brasil Study.^4^ The authors have identified five different patterns, including the “traditional Brazilian”, “non-healthy”, “fast-food/snacks”, “processed” and “healthy”.

The study of eating patterns is extremely complex, as all behavioural factors that contribute to multifactorial chronic disease. They may be influenced by and subject to many confounding factors and interactions, especially with physical activity patterns, cultural patterns, socio-economical variables, gender, urbanisation, food practices, parenting styles and other psychological variables. The authors controlled for some important variables, such as sedentary behaviour and maternal body mass index. It's noteworthy that almost 75% of children presented with sedentary behaviour and almost 60% of mothers had excess weight, thus, the interactions of all these variables must be carefully considered. All these complexities and difficulties in analysing dietary patterns in childhood make this study more valuable, and the discussion must advance further to include other important variables.

The five categories proposed in the study are useful for the purpose of epidemiological studies, but one must be very careful to transpose these categories to clinical recommendations. It is important to highlight that there is a spectrum of healthy to non-healthy habits, thus, naming one of these patterns as healthy, and other as non-healthy may not be so useful clinically, when they are more complex and composed by healthy and unhealthy foods in different proportions.

The traditional Brazilian pattern, for example, is considered healthy by the Brazilian dietary guidelines.^5^ Although the traditional pattern may be not ideal, with a high intake of salt and sugar, the results of the present paper show that the less this pattern was consumed, the greater prevalence of excess weight.

This is very interesting and adds to the knowledge we have already accumulated about Brazilian and other Latin-America traditional dietary patterns. Although for many decades these patterns were overlooked and sometimes considered unhealthy, they were more recently linked with low rates of obesity and chronic diseases.^6^ Of course that many more lifestyle changes have happened simultaneously, but it is very important to consider the relationship between interpretation of previous findings and the marketing of processed foods in these countries, with possible conflicts of interest in industry-funded research. Some decades ago, breastfeeding was considered insufficient feeding for newborn babies and artificial formulae was marketed to paediatricians and families as the most healthy options.^7,8^ The same may have happened to traditional food patterns that are culturally accepted by gradually have been substituted for “modern” processed alternatives.

According to the Brazilian guidelines, the most deleterious pattern is the one that includes mainly ultra-processed foods. This seems to be in accordance with the results that were found in the present study, where the group with a greater prevalence of obesity was the “industrialized group”.

To add to the complexity, the “unhealthy” pattern contains foods that are healthy and recommended for this age group, as the authors describe very well, such as milk, but mostly in unhealthy preparations.

It is very important that we begin to discuss these patterns with more detail to reach better standardisation, allowing international comparisons and a greater understanding of the relations of these patterns with other healthy habits. A recent systematic review pointed out the difficulty of standardisation and the need for a common tool to evaluate dietary intake.^9^

Due to the multifactorial characteristics of childhood obesity, comprehensive interventions that include nutrition education programs and physical activity in multidisciplinary approaches are needed. Evidence is constantly evolving, and guidelines are changing regarding quantitative and qualitative variables, such as certain types of foods or intensity of physical activity. Best results are achieved when multiple actors and scenarios are involved, including family, school, groups, social media, and health professionals and services.^10^
